# Single stem cell gene therapy for genetic skin disease

**DOI:** 10.15252/emmm.201404859

**Published:** 2015-02-27

**Authors:** Jean-Christophe Larsimont, Cédric Blanpain

**Affiliations:** 1 Université Libre de Bruxelles, IRIBHMBrussels, Belgium; 2 WELBIO, Université Libre de BruxellesBrussels, Belgium

## Abstract

Stem cell gene therapy followed by transplantation into damaged regions of the skin has been successfully used to treat genetic skin blistering disorder. Usually, many stem cells are virally transduced to obtain a sufficient number of genetically corrected cells required for successful transplantation, as genetic insertion in every stem cell cannot be precisely defined. In this issue of *EMBO Molecular Medicine*, Droz-Georget Lathion *et al* developed a new strategy for *ex vivo* single cell gene therapy that allows extensive genomic and functional characterization of the genetically repaired individual cells before they can be used in clinical settings.

See also: S Droz-Georget Lathion *et al* (April 2015)

Epidermolysis bullosa (EB) is a family of inherited genetic diseases that are characterized by an extreme skin fragility that causes skin blistering disorder (Carulli *et al*, 2013). This family further divides into four subgroups that arise from different mutations: epidermolysis bullosa simplex, junctional epidermolysis bullosa, dystrophic epidermolysis bullosa (DEB), and Kindler syndrome (Carulli *et al*, 2013). These different genetic disorders result in skin blistering due to the detachment of the epidermis from the dermis or a split within the epidermis (Coulombe *et al*, 2009). DEB can be either dominant or recessive (then called recessive dystrophic epidermolysis bullosa or RDEB), with mutations in gene coding for collagen type VII (*COL7A1*) that localizes to the anchoring fibrils, structures that ensure the adhesion of the basal lamina with the extracellular matrix (Bruckner-Tuderman *et al*, 1999). The disease severely impacts the patient's quality of life and can even cause early lethality in the most severe forms due to defects in skin barrier function leading to infections and development of aggressive skin squamous cell carcinoma (Carulli *et al*, 2013).

Until recently, the only treatment available for EB was restricted to supportive care including pain management, treatment of wound infection and chronic wound, and prevention of mechanical stresses to avoid formation of new blisters.

The important morbidity and mortality associated with EBs stimulated different groups to try unconventional therapies such as type VII collagen administration or grafting of allogeneic fibroblasts with potentially encouraging but not long-lasting results (Carulli *et al*, 2013). In a seminal study, De Luca and colleagues modified the well-established protocol of skin reconstruction used to treat patients with severe burns, developed in the laboratory of Howard Green. In this method, keratinocytes are isolated from skin biopsies and expanded on irradiated fibroblasts, stimulated to differentiate into a functional epidermis before being grafted back to the burnt regions (Gallico *et al*, 1984). Mavillo *et al* cultured the epidermal stem cells of a patient presenting loss-of-function mutation in one of the isoforms of laminin 5, transduced these cells with a retrovirus expressing normal laminin 5, made skin equivalents with the *in vitro* expanded genetically corrected epidermal stem cells, and transplanted sheath of *in vitro* reconstituted skin to the patient. The results were spectacular! The genetically corrected skin presented a long-term functional engraftment with no particular side effects such as skin atrophy or skin cancers after 6.5 years of follow-up (Mavilio *et al*, 2006; Carulli *et al*, 2013). While the authors have analyzed the integration sites of transgene, because the cell population was not clonal, it was not possible to rule out that a minor clonal population of cells within the bulk of the cultured stem cells presented potentially harmful retroviral insertion site, other mutations or chromosome aberrations that were already present in the skin of the patients or induced by the culture conditions.

In this issue of *EMBO Molecular Medicine*, Droz-Georget Lathion *et al* developed a strategy that embraces international standards of good laboratory practices (GLP) and good manufacturing practices (GMP) for medical uses of stem cells and demonstrate the feasibility of single stem cell gene therapy to reconstituted epidermis (Droz-Georget Lathion *et al*, 2015). Barrandon and colleagues used similar approach to the one previously described, the main difference being that individual colonies arising after gene correction of single stem cells were cloned and expanded *in vitro* first.

This approach permitted the study the genomic integrity of the gene-corrected clones of stem cells in great details. They could precisely determine the exact number of copies and integration sites of transgene using LM-PCR and confirmed these observations by fluorescence *in situ* hybridization and next-generation sequencing. Second, this protocol allowed for monitoring the tumorigenic potential of the corrected clones. The authors made the observation that the corrected epidermal stem cells do not form tumor upon grafting into immunocompromised mice, display normal karyotype and normal levels of cell cycle regulators. Finally, the presence of the transgene was assessed by PCR in different organs, which excluded the possibility that genetically engineered epidermal stem cells had disseminated in distant organs.

**Figure 1:**
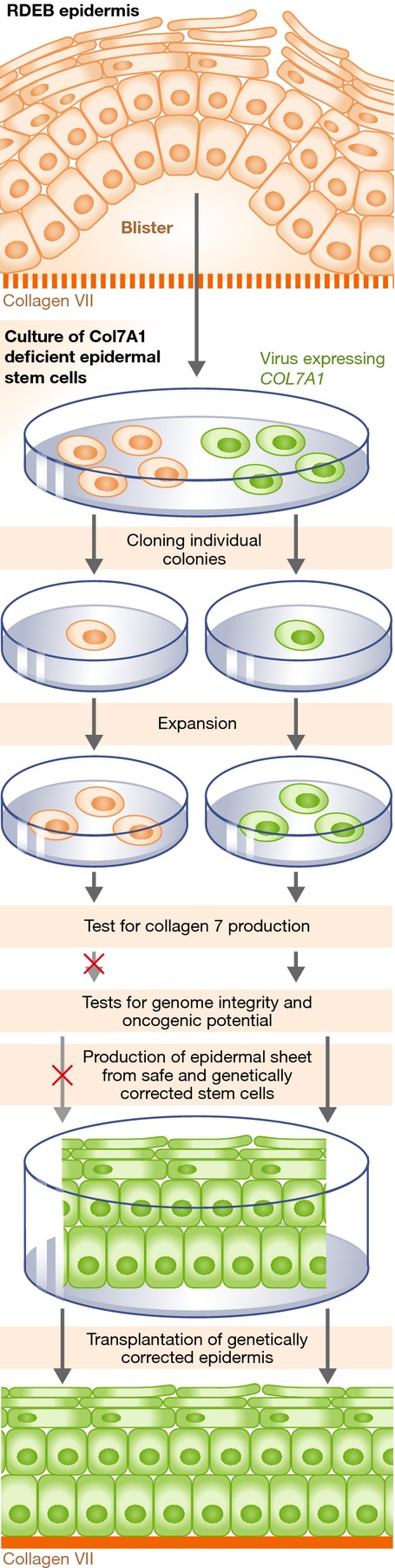
Strategy developed for safe *ex vivo* gene therapy Keratinocyte stem cells from RDEB patient are isolated and infected *ex vivo* with viruses carrying COL7A1 cDNA to correct their function, and colonies arising from a single transduced cell are cloned, selected for their ability to produce collagen 7, and expanded *in vitro*. Their safety is then evaluated by assessing their genomic integrity and oncogenic potential. The clones that expressed Col VII and did not present safety issues are stimulated to reconstruct the epidermis that will be grafted onto the recipient.

In addition, this approach also allowed for a full characterization of the functionalities of the corrected clones. They showed that reconstructed epidermis from keratinocyte stem cells transduced with the normal form of the defective gene could form an epidermis that engrafted onto immunocompromised mice and was able to produce functional collagen VII that localized to the basal lamina and corrected the blistering phenotype of RDEB.

Overall, the study of Droz-Georget Lathion demonstrates the feasibility of *ex vivo* single cell gene therapy that allows a careful characterization of the recombined cells prior to medical use. However promising, this study was performed on epidermal stem cells from a single patient and it will be necessary to reproduce this finding in other patients, and independently of the severity of the disease since this aspect might represent a limiting factor for obtaining a sufficient amount of epidermal stem cells that can grow and expand *in vitro* (Mavilio *et al*, 2006). Furthermore, it would be of great interest to evaluate the scalability of this single stem cell gene therapy for more applications to the treatment of other genetic diseases affecting other tissues. Novel strategy using patient-derived iPS cells in which Col7 has been genetically corrected, which are then differentiated into stratified epidermis and transplanted into the damaged site, appears as an interesting alternative for the treatment of genetic skin disorders (Sebastiano *et al*, 2014; Umegaki-Arao *et al*, 2014; Wenzel *et al*, 2014).
